# Evaluating knowledge and awareness of cervical cancer, human papillomavirus and human papillomavirus vaccination among women in East London, South Africa

**DOI:** 10.4102/phcfm.v18i1.5261

**Published:** 2026-05-19

**Authors:** Tabita Boto, Anthony M. Feketshane, Nondumiso Ngxola

**Affiliations:** 1 Department of Obstetrics and Gynaecology, Faculty of Medicine and Health Sciences, Walter Sisulu University, East London, South Africa

**Keywords:** human papillomavirus, HPV vaccine, cervical cancer, awareness, knowledge, East London, South Africa, women’s health

## Abstract

**Background:**

Cervical cancer poses a major health risk for women in low- and middle-income countries, with persistent human papillomavirus (HPV) types 16 and 18 as leading causes. In South Africa, screening rates are low, and awareness and vaccine uptake remain insufficient despite a national immunisation programme since 2014.

**Aim:**

This study evaluated women’s knowledge and awareness of cervical cancer, HPV and HPV vaccination at three healthcare levels in East London, South Africa, and examined demographic factors and differences between settings.

**Setting:**

The study was conducted at five public health facilities in East London, Eastern Cape Province, South Africa.

**Methods:**

An analytical cross-sectional study was conducted. Three hundred and five female participants completed questionnaires on cervical cancer, HPV and vaccination. Data were analysed with STATA 17; knowledge was scored and classified from ‘very poor’ to ‘excellent’, and statistical tests determined associations.

**Results:**

The median knowledge score was 28.6%. Awareness rates were as follows: cervical cancer (48.5%), HPV (25.9%) and HPV vaccination (29.2%). Community health centre attendees had the lowest scores. Only 6.2% had ‘excellent’ knowledge, and 52% had ‘poor’ or ‘very poor’ knowledge. Higher education and hospital-based care correlated with better scores (*p* < 0.001). Vaccine uptake among eligible female relatives was only 12%.

**Conclusion:**

Knowledge and awareness of cervical cancer, HPV and vaccination are low, especially in primary care. Pap smear usage is high, but understanding remains limited. Improved community education and provider involvement are needed to boost awareness and vaccine acceptance if South Africa is to meet the World Health Organization cervical cancer targets by 2030.

**Contribution:**

This study provides current evidence on HPV-related knowledge gaps among women in the Eastern Cape and highlights the need for targeted interventions within primary health care settings.

## Introduction

Cervical cancer remains one of the most preventable malignancies, yet it continues to rank as the fourth most common cancer in women worldwide^1^ after breast, colorectal and lung cancers.^2^ In 2020, an estimated 604 127 new cases of cervical cancer in women^3^ and 341 831 deaths were reported globally, with the majority occurring in low- and middle-income countries (LMICs).^4^ Sub-Saharan Africa (SSA) carries a disproportionate burden: the age-standardised incidence rate is among the highest globally, with approximately 22 new cases and 14 deaths per 100 000 women annually according to GLOBOCAN (Global Cancer Observatory) 2022 STATS. In Africa alone, around 126 000 women are diagnosed annually, and more than 50 000 women die from the disease.^3^ The situation in South Africa mirrors this crisis. The country records approximately 10 702 new cervical cancer cases each year (estimations for 2020), ranking as the second most common cancer among women and the leading gynaecological malignancy.^3,4^ Among women aged 15–44 years, it is the most prevalent cancer. Data from Frere Hospital in the Eastern Cape show that cervical cancer accounted for 78% of all gynaecological cancers treated between 2017 and 2021.^5^ Human papillomavirus (HPV) infection is the primary cause of cervical cancer worldwide.^6^ High-risk HPV subtypes, particularly HPV-16 and HPV-18, are responsible for around 90% of cases. Harald zur Hausen confirmed this causal link as far back as early 1980s, shifting global strategies towards HPV vaccination as a cornerstone of prevention.^7^ Despite this, other risk factors such as human immunodeficiency virus (HIV) co-infection, smoking, high parity and long-term oral contraceptive use exacerbate the risk, particularly in regions like South Africa with high HIV prevalence.^8^ The World Health Organization (WHO)^9^ launched its 90-70-90 strategy in 2020, aiming to eliminate cervical cancer as a public health problem by 2030. The targets include vaccinating 90% of girls fully against HPV by age 15 years; screening 70% of women at ages 35–45 years; and treating 90% of women diagnosed with cervical disease. Achieving this requires not only access to vaccines and screening, but also strong public awareness and acceptance. South Africa introduced a school-based HPV vaccination programme in 2014, targeting girls aged 9–13 years in public schools. While this has increased vaccine coverage nationally, uptake remains uneven. Barriers include limited community awareness, misinformation, socio-cultural factors and disparities between rural and urban settings.^10^ Evidence indicates that knowledge about HPV and cervical cancer prevention remains poor even among women accessing healthcare services.^11,12^ This knowledge gap undermines vaccine uptake, timely screening and overall cancer prevention.

This study therefore aimed to evaluate women’s knowledge and awareness of cervical cancer, HPV infection and HPV vaccination at three levels of healthcare in East London. By examining demographic predictors of knowledge and differences between primary, secondary and tertiary healthcare settings, the study sought to identify gaps that can inform public health education and support progress towards national and global elimination goals.

## Research methods and design

### Study design and setting

An analytical cross-sectional study was conducted between 09 January 2025 and 30 January 2025 across five public health facilities in Buffalo City Municipality, East London, Eastern Cape Province, South Africa. These included two community health centres (CHC 1 and CHC 2), two gynaecology outpatient departments (GOPD) (Hospital A and Hospital B), and the oncology unit at Hospital A.

### Study population and sampling

#### Inclusion criteria

All women and escorts (aged between 25 years and 65 years) attending the selected facilities during the study period were approached and invited to participate. Those who consented were enrolled.

#### Exclusion criteria

Women younger than 25 years and those too sick to partake were excluded. The sampling method was non-probability purposive sampling, with participants self-selecting into the study.

The sample size was calculated based on an estimated prevalence of 20% – 35% awareness of HPV in the general population.^13,14^ To achieve adequate power, a minimum of 250–350 respondents was required. Allowing for a 10% non-response rate, 278–389 individuals were targeted. Ultimately, 305 participants were recruited for the study.

### Data collection

A structured questionnaire, available in English and isiXhosa, was administered by the researcher and assistants. Sections of the questionnaire included demographics, cervical cancer awareness, HPV awareness, and HPV vaccine awareness. Participants were guided through each item.

### Knowledge scoring

Thirteen knowledge items were scored, with one point for each correct response. Multi-response items allocated fractional points. Two additional items assessed awareness of the HPV vaccine. Total knowledge was calculated as a percentage (0% – 100%), categorised as: excellent (> 80%), good (60% – 80%), moderate (40% – 59%), poor (20% – 39%) and very poor (< 20%).

### Data analysis

Data were entered into Microsoft Excel by the researcher and analysed using STATA version 17 (StataCorp, College Station, Texas, United States [US]) by a biostatistician. Descriptive statistics were presented as frequencies, percentages, and medians with interquartile ranges. Chi-square and Kruskal–Wallis tests were applied for group comparisons.

### Ethical considerations

Ethical approval to conduct this study was obtained from Walter Sisulu University (Ref: 183/2024) and the Eastern Cape Health Research Committee (Ref: EC_202411_009). Written consent was obtained from all participants.

## Results

### Sociodemographic characteristics

From the sample of 305 participants, 85% of the women were aged 30 years or more, 40% had a daughter aged 9–22 years, and almost two-thirds (64%) had Grade 7–12 as their highest education level (Table 1).

**TABLE 1:** Sociodemographic characteristics of the sample (***N*** = 305).

Sociodemographics	Summary	
	*n*	%
***N***	305	-
Institution		
Hospital B GOPD	62	20.3
CHC 1	60	19.7
Hospital A GOPD	60	19.7
CHC 2	63	20.7
Oncology	60	19.7
Level of institution		
CHC	123	40.3
OPD	122	40.0
Oncology	60	19.7
Age (years)		
< 30	45	14.8
30+	260	85.2
Level of education		
None	5	1.6
Grade 0–7	46	15.1
Grade 7–12	196	64.3
Tertiary	58	19.0
Residence		
Amathole	24	7.9
BCM	238	78.3
Chris Hani	16	5.3
Joe Gqabi	2	0.7
Other	24	7.9
Marital status		
Divorced	11	3.6
Married	98	32.1
Other	5	1.6
Single	183	60.0
Widowed	8	2.6
Children		
None	54	17.7
One or more	251	82.3
Daughters (aged 9–22 years)		
Yes	121	39.7
No	184	60.3

CHC, community health centre; OPD, out patients department; GOPD, Gynae Out Patient Department; BCM, Buffalo City Municipality.

### Knowledge and awareness of cervical cancer, human papillomavirus and human papillomavirus vaccination

Awareness of cervical cancer was 48.5%; HPV awareness was 25.9%; and HPV vaccine awareness was 29.2%. Only 12% of eligible relatives had received the HPV vaccine (Table 2).

**TABLE 2:** Comparisons of knowledge and awareness across levels of institution (***N*** = 305).

Awareness and knowledge	Level of institution								
	CHC		OPD		Oncology		Total		Test
	*n*	%	*n*	%	*n*	%	*n*	%	
*N*	123	40.3	122	40.0	60	19.7	305	100.0	-
**CC awareness**	-	-	-	-	-	-	-	-	< 0.001
No	91	74.0	47	38.5	19	31.7	157	51.5	-
Yes	32	26.0	75	61.5	41	68.3	148	48.5	-
**HPV awareness**	-	-	-	-	-	-	-	-	0.002
No	104	84.6	80	65.6	42	70.0	226	74.1	-
Yes	19	15.4	42	34.4	18	30.0	79	25.9	-
**HPV vaccine awareness**	-	-	-	-	-	-	-	-	0.313
No	93	75.6	83	68.0	40	66.7	216	70.8	-
Yes	30	24.4	39	32.0	20	33.3	89	29.2	-

Note: Total knowledge score%: CHC –21.4% (14.3% – 35.7%), OPD – 35.7% (28.6% – 64.3%), Oncology – 35.7% (28.6% – 64.3%), Total – 28.6% (21.4% – 42.9%), Test – < 0.001. Knowledge CC%: CHC – 50.0% (33.3% – 66.7%), OPD – 66.7% (50.0% – 83.3%), Oncology – 83.3% (58.3% – 83.3%), Total – 66.7% (50.0% – 83.3%), Test – < 0.001. Knowledge HPV%: CHC – 0.0% (0.0% – 50.0%), OPD – 0.0% (0.0% – 50.0%), Oncology – 0.0% (0.0% – 50.0%), Total – 0.0% (0.0% – 50.0%), Test – 0.119.

CHC, community health centre; HPV, human papillomavirus; OPD, Out Patient Department; CC, cervical cancer.

### Sociodemographic comparisons

Significant associations were found between education, residence and awareness levels (Table 3).

**TABLE 3:** Comparisons of sociodemographic variables across levels of institution (*N* = 305).

Sociodemographic variables	Level of institution								
	CHC		OPD		Oncology		Total		Chi^2^ Test
	*n*	%	*n*	%	*n*	%	*n*	%	
*N*	123	40.3	122	40.0	60	19.7	305	100.0	-
Institution	-	-	-	-	-	-	-	-	-
Hospital B GOPD	0	0.0	62	50.8	0	0.0	62	20.3	< 0.001
CHC 1	60	48.8	0	0.0	0	0.0	60	19.7	-
Hospital A GOPD	0	0.0	60	49.2	0	0.0	60	19.7	-
CHC 2	63	51.2	0	0.0	0	0.0	63	20.7	-
Oncology	0	0.0	0	0.0	60	100.0	60	19.7	-
Age (years)	-	-	-	-	-	-	-	-	0.002
< 30	14	11.4	28	23.0	3	5.0	45	14.8	-
30+	109	88.6	94	77.0	57	95.0	260	85.2	-
Level of education	-	-	-	-	-	-	-	-	0.005
None	0	0.0	2	1.6	3	5.0	5	1.6	-
Grade 0–7	27	22.0	14	11.5	5	8.3	46	15.1	-
Grade 7–12	67	54.5	88	72.1	41	68.3	196	64.3	-
Tertiary	29	23.6	18	14.8	11	18.3	58	19.0	-
Residence	-	-	-	-	-	-	-	-	< 0.001
Amathole	2	1.6	16	13.2	6	10.0	24	7.9	-
BCM	119	96.7	91	75.2	28	46.7	238	78.3	-
Chris Hani	0	0.0	7	5.8	9	15.0	16	5.3	-
Joe Gqabi	0	0.0	0	0.0	2	3.3	2	0.7	-
Other	2	1.6	7	5.8	15	25.0	24	7.9	-

CHC, community health centre; OPD, out patients department; GOPD, Gynae Out Patient Department; BCM, Buffalo City Municipality.

## Discussion

This study explored the knowledge and awareness of cervical cancer, HPV infection and the HPV vaccine among women attending different levels of healthcare in East London. The findings highlight substantial gaps, especially at the primary care level and reflect systemic challenges in public health education and preventive health engagement. While 76.4% of participants had undergone a Pap smear, only 48.5% were aware that cervical cancer is preventable, and just 41.6% correctly identified HPV as a causative agent. These results suggest that cervical screening may be occurring without adequate understanding of its purpose. This aligns with findings in China, where women attending a hospital for routine gynaecological care had poor knowledge of cervical cancer and its risk factors, despite good screening rates.^15^ Similarly, a South African study by Ngcobo et al.^11^ found that while access to screening services is improving, knowledge and uptake are still uneven, with women in rural and resource-limited areas at a significant disadvantage. Only 26% of participants had heard of HPV, and 29% had heard of the HPV vaccine. These figures are consistent with international findings. For instance, a systematic review by Taebi et al.^14^ in Iran highlighted that women had low awareness of HPV and the available vaccines, even in urban hospital settings. Likewise, a study conducted in Serbia found good HPV knowledge among women attending gynaecology clinics,^13^ but limited awareness of the vaccine. In contrast, Harrison et al.^16^ reported significantly higher HPV awareness among parents in the southern United States, partly attributed to structured school-based vaccine promotion and public health campaigns. The disparity underscores the impact of national-level educational and policy interventions. Notably, 79% of those who had heard of HPV in our study learned about it from health professionals. This finding was mirrored in other LMIC settings,^12^ confirming the vital role of frontline healthcare workers in patient education. Knowledge scores were significantly lower among women attending CHC facilities compared to those at OPD or oncology units. This trend has been reported in other African studies, where proximity to tertiary care is associated with better health literacy because of higher exposure to health professionals and structured health education programmes.^17^ Our findings reinforce that women attending primary health care facilities, who make up the bulk of the population, remain underserved in terms of health education, despite being the ideal target for preventive interventions like vaccination and early screening. Only 12.2% of participants reported a female relative aged 9–18 years who had received the HPV vaccine. This is below the national average reported by Amponsah-Dacosta et al.,^10^ who found that 61% of South African girls had received the full vaccine schedule in public schools by 2020. This discrepancy may reflect geographical and socioeconomic barriers to vaccine access in the Eastern Cape, as well as inconsistent follow-up after initial vaccine administration. Moreover, vaccine hesitancy and misinformation remain pervasive. Smolarczyk et al.^7^ emphasised that low vaccine coverage in LMICs is often driven by a lack of awareness, cultural myths and mistrust in health systems. These themes were echoed in responses from participants in this study. The WHO’s strategy to eliminate cervical cancer as a public health problem by 2030 is built on achieving the 90-70-90 targets: 90% of girls fully vaccinated with the HPV vaccine by age 15 years, 70% of women screened with a high-performance test by ages 35 years and 45 years and 90% of those diagnosed with cervical disease receiving appropriate treatment. The findings of this study reveal significant gaps that threaten the achievement of these targets in the Eastern Cape region. With only 29% vaccine awareness and 12% of participants reporting that a female relative had received the vaccine, we are far below the 90% vaccination goal. Although Pap smear uptake was relatively high (76.4%), actual understanding of cervical cancer and its link to HPV remains low, suggesting that screening is not always linked to informed health-seeking behaviour. Without increased efforts to improve HPV education, these goals will remain out of reach.

### Limitations

The study would have shown a clearer reflection of knowledge in our society if it were conducted on women from their homes instead of health care institutions. However, the mere fact that even these women who seek assistance at health care centres lacked knowledge on the subject shows just how poor the results would have been if the questionnaires were posed on women not attending healthcare services. The type of study is also a limitation as we cannot elucidate determinants. Therefore, the study is establishing only an association and therefore generating only a hypothesis.

### Strengths

The researcher herself conducted the study, and women were assisted in completing the questionnaire, limiting errors and ensuring that all questions were answered. The questionnaire was in the women’s mother tongue, making it easy to understand and complete. The sample size was large enough to cover a wide variety of women from a vast geographical area.

## Conclusion

This study revealed low levels of knowledge and awareness of cervical cancer, HPV and HPV vaccination in East London, particularly at the primary care level. Although Pap smear uptake was relatively high, understanding of its purpose and link to cancer prevention was inadequate. Without urgent educational interventions, South Africa is unlikely to meet the WHO targets of eliminating cervical cancer by 2030.

### Recommendations

- Strengthen health education at the primary level: This should be done in CHC facilities, where the lowest knowledge levels were observed. They need structured, frequent health education sessions focused on cervical cancer, HPV and the benefits of vaccination.
- Train and empower health workers: Because most women who knew about HPV received this information from health professionals, more investment in training assistant nurses, registered nurses, community health workers and doctors to offer consistent health messaging during consultations is crucial.
- Community outreach and school engagement: Efforts must be made to extend HPV vaccine awareness beyond the school system, targeting parents and caregivers through local media, churches, community meetings and women’s groups.

### Key messages for policy and practice

- Cervical cancer is the leading gynaecological malignancy in South Africa.
- Awareness of HPV and HPV vaccination is critically low in East London.
- Human papillomavirus vaccine uptake remains poor, far below the WHO targets.
- Healthcare workers are central to patient education but require further training and support.
- Stronger community engagement and targeted education are essential to eliminate cervical cancer as a public health problem.
